# Effect of Graphic Cigarette Warnings on Smoking Intentions in Young Adults

**DOI:** 10.1371/journal.pone.0096315

**Published:** 2014-05-07

**Authors:** Hart Blanton, Leslie B. Snyder, Erin Strauts, Joy G. Larson

**Affiliations:** 1 Department of Psychology, University of Connecticut, Storrs, Connecticut, United States of America; 2 Department of Communication Sciences, University of Connecticut, Storrs, Connecticut, United States of America; 3 Center for Health, Intervention, and Prevention, University of Connecticut, Storrs, Connecticut, United States of America; Newcastle University, United Kingdom

## Abstract

**Introduction:**

Graphic warnings (GWs) on cigarette packs are widely used internationally and perhaps will be in the US but their impact is not well understood. This study tested support for competing hypotheses in different subgroups of young adults defined by their history of cigarette smoking and individual difference variables (e.g., psychological reactance). One hypothesis predicted adaptive responding (GWs would lower smoking-related intentions) and another predicted defensive responding (GWs would raise smoking-related intentions).

**Methods:**

Participants were an online sample of 1,169 Americans ages 18–24, who were randomly assigned either to view nine GWs designed by the FDA or to a no-label control. Both the intention to smoke in the future and the intention to quit smoking (among smokers) were assessed before and after message exposure.

**Results:**

GWs lowered intention to smoke in the future among those with a moderate lifetime smoking history (between 1 and 100 cigarettes), and they increased intention to quit smoking among those with a heavy lifetime smoking history (more than 100 cigarettes). Both effects were limited to individuals who had smoked in some but not all of the prior 30 days (i.e., occasional smokers). No evidence of defensive “boomerang effects” on intention was observed in any subgroup.

**Conclusion:**

Graphic warnings can reduce interest in smoking among occasional smokers, a finding that supports the adaptive-change hypothesis. GWs that target occasional smokers might be more effective at reducing cigarette smoking in young adults.

## Introduction

The Family Smoking Prevention and Control Act of 2009 gave the Food and Drug Administration (FDA) the power to mandate inclusion of graphic warnings (GWs) in cigarette advertising and packaging. In response, the FDA developed a set of 9 GWs it sought to embed on all cigarette packaging in the US. After the 6^th^ Circuit Court of Appeals upheld the mandate in 2011, the Court of Appeals for the Washington DC Circuit ruled against it in 2012. The constitutionality of this legislation will likely be decided by the Supreme Court in the future, but in March, 2013, the FDA indicated that it would not ask the Court to review the federal appeal. By not challenging the ruling, the FDA avoided the possibility of a defeat that could have effectively ended government efforts to place GWs on cigarette packaging. Although one cannot be certain of the legal standard that the courts will apply in evaluating future efforts, one likely analysis hinges on whether GWs are judged to “directly advance” the government's interests in protecting Americans from the negative health effects of smoking [1]. This standard was applied by the DC Circuit, which argued that “The FDA has not provided a shred of evidence … showing that the graphic warnings will ‘directly advance’ its interest in reducing the number of Americans who smoke.” Some legal scholars believe that, if this standard is applied by the Supreme Court, “even without evidence of the impact of graphic warnings on behavior, strong evidence that they affect behavioral intent, and that intent predicts behavior, should be sufficient for the warnings to be upheld” [2] (p. 334). The current project speaks to this legal standard by utilizing experimental methods to examine the effects of GWs on behavioral intention. It expands prior research by critically examining the role that smoking history and individual differences have as factors that might moderate the effect of GW exposure on behavioral intention. We explore this question using a community sample of young adults, as this is the age group in which smoking is most prevalent [3] and so it also the group that is most likely to encounter GWs on cigarette packaging.

### Current Evidence

Many tobacco researchers have advocated for the use of GWs as an important step in reducing smoking rates [4]–[8]. This position is supported by evidence that smoking rates have decreased in countries that have mandated GWs [4], [9]–[11]. Naturalistic investigations of this kind can provide insights into the potential influence of GWs in the US, but they can be limited in two important respects. First, in terms of legal standards, courts have at times have been hesitant to draw strong inferences from data generated in countries other than the US [2], a concern expressed by The DC Circuit. If international data are similarly dismissed or viewed critically in future decisions, data on behavioral intention may be the FDA's best option to establish a government interest. Second, in terms of empirical standards, naturalistic reports have limited ability to document the influence of individual difference factors. Even if GWs have reduced smoking in other countries, national trends might hide unwanted or unintended effects. As early as 1976, Rogers and Mewborn documented that exposure to antismoking communications can have the unintended consequence of increasing smoking-related intentions, an effect now commonly referred to as a “boomerang effect” [12]. It is on this basis that some have argued against GW mandates [13]. Through aggregate reporting, naturalistic investigations could fail to detect subgroups for which such boomerang effects can be anticipated.

Experimental studies can provide the precision needed to identify individual difference factors that alter the influence of GWs on intention, but the current literature has given limited attention to this question. This is due in part to a practical constraint: many experiments employ sample sizes that lack the statistical power needed to test for the moderating effects of individual difference factors [14]–[16]. Compounding this limitation, the majority of studies have focused on one of two different classes of dependent variables. One class relates to self-reported reactions to GWs, with research identifying factors that increase message acceptance, perceived effectiveness and believability [17]. Research of this type is critical to the design of impactful GWs, but it also is limited in that research participants often are unable to identify the factors that will influence their actual behaviors [18].

A second class of variables relates to the beliefs and expectations that might be shaped by GWs. Research of this kind suggests that GWs can increase perceptions of the harm of smoking [19]–[21], decrease the desirability of the smoking social image [22], lower the desirability of cigarette purchases [23], increase negative affective reactions to smoking cues [24] and improve recall for other health information [25], [26]. These changes all suggest that GWs will promote more negative and less positive attitudes towards smoking. However, changes in attitudes do not always translate into changes in behavioral intention, and modern attitude-behavior models assume this step is critical for attitudes to influence behavior [27]. At present, evidence for the influence of GWs on intention is mixed. Some research suggests GWs decrease smoking intentions [14], [15], some has documented null or inconsistent effects [28], and some has revealed evidence of boomerang effects, whereby exposure to GWs increases smoking intentions [16]. The equivocal nature of experimental research on behavioral intention points to the need for studies that utilizes larger sample sizes, to determine if variability in smoking history can account for some the heterogeneity found in the published literature.

### Smoking History

Young adults differ considerably in their experience with cigarettes, and so it seems unlikely that GWs will exert uniform influence on all members of this age group, independent of their smoking experiences. The majority of young adults who have not tried a cigarette will never initiate this action [3]. This suggests that, on the whole, one should expect floor effects in younger adults who have never smoked a cigarette. This group has relatively low risk of smoking, and so GWs might have little ability to further suppress behavioral intentions. The same might be expected for young adults who have smoked in the past but who have successfully transitioned into inactive smokers. Exposure to GWs might also be of limited use to those who have become daily smokers – but for different reasons. A simple image-based intervention lacks the components typically required to promote smoking cessation in daily smokers [29], and so it seems unlikely that GWs alone can dent the strong intention to smoke among daily smokers cf., [30].

This analysis suggests that GW effects on young adults might be concentrated among those who occupy the middle range of smoking experience – those who have smoked in the past but have not transitioned into daily smokers. However, this “middle range” is a heterogeneous group [31]. Some young adults who smoke occasionally are in the early stages of experimenting, whereas others are in a stable pattern of intermittent use [32], [33]. This analysis suggests that GW effects will be concentrated in those who do smoke but do so less than daily. However, it is important to note that two forms of intention are predictive of smoking decisions. One pertains to the willingness to smoke in situations that promote smoking [34], [35] and the other to the more proactive intention to quit [36]. Occasional smokers who are in the early stages of trying cigarettes may not have developed identities as smokers, and so they might not feel they need to “quit” a behavior they don't believe they have begun [37]. The more they have smoked, however, the less they can engage in that logic. This suggests that GWs might shift from primarily diminishing willingness to smoke in the future among occasional smokers that are in early stages of smoking, to influencing intentions to quit smoking among those who are in a more stable patter of occasional tobacco use.

#### Adaptive versus Defensive Responding

The above predictions follow from an *adaptive-response hypothesis*, as they assume that those in the middle group are largely open and responsive to the information presented by GWs, though in different ways based on their past experiences. Running contrary to these assumptions are predictions that follow from a *defensive-response hypothesis*. This perspective on GWs is based on a large body of experimental research indicating that individuals who have engaged in risky behaviors in the past tend to react more defensively to risk-prevention messages [38]–[42]. In the domain of smoking, for instance, research suggests that smokers disengage from antismoking messages more than nonsmokers [43] and are more critical of message content [44]. In at least some instances, defensive reactions to prevention messages can be so strong as to produce boomerang effects [45]. It is based on findings such as these that some have argued against GW mandates [13].

It is unclear which groups might be most prone to reacting defensively, but the general prediction would be a stronger boomerang effects among those who would be most clearly threatened by GW message content. This might point to daily smokers as the likely candidates, although this group could evidence a “ceiling effect,” in that their smoking intention can go no higher than the normal baseline. Among occasional smokers, however, defensive reactions might be more pronounced the more cigarettes smoked over the course of their lifetime. It is these more active occasional smokers that could feel most directly criticized by antismoking GWs. Alternatively, boomerang effects might be linked, not to smoking history, but to individual difference factors that are predictive of defensive message processing. A review of the literature suggests three strong possibilities: (1) *psychological reactance*, which can cause individuals to resist negatively framed messages that are perceived as overly coercive [46], [47], (2) *self-esteem*, which can cause individuals to become more committed to risky behaviors in the face of critical health information [48], [49], and (3) *sensation seeking*, as a predictor of more fearful responding to social stimuli [50], [51], which might lead to a documented “fear control” response that can promote defensive rejection of prevention messages [52], [53]. The current study critically examined the adaptive-response and defensive-response hypotheses, by testing if the effect of GW exposure on smoking-related intentions was moderated, either by smoking history or by any of these three individual difference factors.

### Summary of Hypotheses

We predict that GW exposure will only influence smoking-related intentions for certain subgroups of young adults, and we test competing predictions for the patterning of these effects. If adaptive response predictions hold, GW effects will be concentrated in the group of occasional smokers, although it might differentially target willingness to smoke in the future versus the intention to quit, based on severity of past smoking history. If defensive response predictions hold, GW exposure will cause boomerang effects, with stronger effects observed either in groups that have engaged in heavier smoking (i.e., occasional smokers who have longer lifetime histories of smoking and possibly daily smokers) or in those with personal characteristics that promote rejection of risk-prevention messages (e.g., psychological reactance).

## Materials and Methods

### Ethics Statement

The protocol for this study, including online recruitment, informed consent, and data collection, was approved by the Institutional Review Board at the University of Connecticut. All data and materials included in this report will be made available upon request to the first author.

### Overview

Participants (1) completed a questionnaire assessing smoking history and smoking-related willingness and intentions, (2) were randomly assigned to an experimental condition that viewed the 9 FDA graphic warning labels or to a control condition that did not view these labels, and then (3) completed a questionnaire assessing post-manipulation smoking-related willingness and intentions.

### Participants

This study used an online presentation of experimental stimuli and collection of data, a method that has been used in the past to study reactions to GWs [54]. Participants were recruited via Amazon's Mechanical Turk, an internet crowd-sourcing site that can be used to connect interested participants with psychology researchers for pay. Evidence suggests that this portal replicates experimental effects obtained in laboratory settings, but it does so with samples that are more diverse than college samples [55]. Participants were paid $1.00 to complete a questionnaire that was designed to take 30–45 minutes. From an original sample of *N* = 1648 respondents, 268 were eliminated for not completing the questionnaire, 192 for not living in the US or being outside the age range and 19 for listing inconsistent responses on the questionnaire.

This resulted in a sample of *N* = 1169 US Citizens, aged 18–24. Respondents were 50% female and slightly skewed to the older end of the range (Mean = 21.75; Median = 22). Compared to November 2012 census population estimates of 18–24 year olds [56], the sample included more Whites (79.3% vs 74% nationally) and Asian or Asian Americans (9.5% vs 5.0%), and fewer African Americans (7.4% vs 15.9%) and Hispanic/Latinos (7.4% vs 20.9%). (Multiple responses allowed and so values do not add to 100%.) The sample was more educated than census estimates for this age group. (For highest education level, 4% had a graduate degree vs 1% for the U.S; 25% had bachelor's degree vs 8% for the U.S.; 8% had an associate's degree vs 5% for the U.S.; 49% had some college vs 41% for the U.S.; 12% finished high school vs 29% for the U.S.; 2% had some high school vs 16% for the U.S.; [57]). Analysis (available upon request) failed to identify moderating effects of age, sex, ethnicity or education on the experimental effects reported here.

### Stimulus Presentation

Those in the experimental condition were informed about the FDA decision to require graphic warning labels on cigarette packaging in the US (which was still in effect at the time of the study), after which they were exposed to all 9 FDA images. Participants first viewed the images three at a time, embedded on generic cigarette packages. While viewing each set of 3 images, participants were asked to rate which of the three pictures and written warnings they found to be the most attention-grabbing, after which they were shown all 9 warning labels again on a single screen shot and asked to rate which was most memorable. Attention ratings were not of interest but were included to ensure that participants took a moment to examine the labels on the packages. Those in the control condition were informed of the FDA plans to place images on cigarette packaging, but they were not shown the actual images.

### Measures

#### Smoking history

In order to capture the full range of meaningful differences documented in this age group, smoking history was operationalized both in terms of lifetime and recent history of smoking [33], [37]. *Lifetime smoking history* was assessed with the question, “About how many cigarettes have you ever smoked?” with responses made on a 7-point response scale that ranged from “I have never smoked” to “100 or more.” These responses were used to create three categories of participants: (1) those with *no experience*, who had never smoked (*n* = 538), (2) those with *moderate experience*, who had smoked in the past but had fewer than five packs (100 cigarettes) in their lifetime (*n* = 319), and (3) those with *heavy experience*, who had smoked 100 or more cigarettes in their lifetime (*n* = 312). *Recent smoking history* was assessed by having participants list the number of days out of the previous 30 they had smoked “even 1 or 2 puffs” of a cigarette. These responses were used to create three cross-cutting categories of participants: (1) those who were currently *inactive*, who had not smoked in the prior 30 days (*n* = 834), (2) those who were *occasional smokers*, who had smoked some but not all of the prior 30 days (*n* = 210), and (3) those who were *daily smokers*, who had smoked all of the prior 30 days (*n* = 125). A cross-tabulation of these two variables (Table 1) reveals that participants with moderate lifetime smoking history could be further delineated as either inactive or occasional smokers, whereas those with heavy lifetime smoking history could be further delineated as inactive, occasional or daily smokers.

**Table 1:** Smoking Profiles.

	Lifetime Smoking history			
	*None*	*Moderate*	*Heavy*	Totals
Recent Smoking history				
*Inactive*	538	244	52	834
*Occasional*	0	75	135	210
*Daily*	0	0	125	125
Totals	538	319	312	1169

*Note*: Each cell reports sample size for that group. For lifetime smoking history, “none” indicates having never smoked, “moderate” indicates having smoked but fewer than 100 cigarettes, and “heavy” indicates having smoked 100 or more in a lifetime. For recent smoking history, “inactive” indicates having not smoked in the previous 30 days, “occasional” indicates having smoked in some but not all of the previous 30 days, and “daily” indicates having smoked all of the previous 30 days.

#### Behavioral Intention

Both *smoking intention* and *quit intention* were assessed before and after the experimental manipulation. In each case, the wording of the questions and the response scales provided were altered after the experimental manipulation to prevent participants from simply repeating pre-manipulation responses at post-manipulation assessment (which would artificially suppress treatment effects). Smoking intention was measured prior to the manipulation with two questions; one assessing smoking expectation (“Do you think you might smoke a cigarette sometime soon?”) and another assessing smoking willingness (“If a friend offered you a cigarette, would you think about smoking it?”). Both were reported using a 5-point scale that ranged from “definitely yes” to “definitely no” (*alpha* = 0.91). Smoking intention was measured again after the manipulation with two questions (“If it would help me go with the flow at a party with friends, I would be willing to smoke a cigarette” and “In the next 30 days, how likely are you to take a puff if a friend offered you a cigarette?”), using a 7-point scale that ranged from “strongly disagree” to “strongly agree” (*alpha* = 0.96). *Quit intention* was measured prior to the manipulation with two questions (“To what extent do you plan to stop smoking in the very near future?” – using a 4-point scale that ranged from “not at all” to “extremely” – and “Choose the number that indicates how you think about stopping smoking at present” – using an 11-point scale that ranged from 0 = “I have not thought about stopping” to 10 = “I am taking action to stop smoking”) (*alpha* = 0.52). Quit intention was measured post-manipulation with five questions (e.g., “It is important to me to stop smoking at some point in the near future”), using a 7-point scale that ranged from “strongly disagree” to “strongly agree” (*alpha* = 0.80).

#### Individual Differences

Prior to the experimental manipulation, participants also completed three individual difference measures theorized to be predictive of defensive processing of risk-prevention messages: *psychological reactance* [58], *self-esteem* [59] and *sensation seeking* [60].

### Analytic Approach

The cross-tabulation of recent and lifetime history shown in Table 1 presented an analytic challenge. Because there are missing cells that do not or cannot contain data (e.g., individuals who have not smoked in their lifetime but who have smoked recently), it was not possible to investigate how these lifetime and recent smoking history might interact with one another and with GW-exposure in the prediction of behavioral intention. To address this, we employed an analytic approach in which the effects of the two smoking history variables were examined in two steps.

The *first step* applied standard analysis of variance (ANOVA) methods to the entire data set to assess the independent and interactive effects of lifetime smoking history and GW-exposure on behavioral intention, after which simple main effects tests were conducted to determine if the effects of GW-exposure were concentrated within groups defined by lifetime smoking history [61]. The *second step* employed exploratory simple main effects analysis. These were focused on determining if any effects of GW-exposure were further concentrated within subgroups defined on the basis of their recent smoking history. One analysis was conducted on the subgroup with moderate lifetime smoking history and another was conducted on the subgroup with heavy lifetime smoking history. Each time, analyses tested for simple main effects of GW-exposure for different groups defined by their recent smoking history.

This two-step approach thus made it possible to determine if the effects of GW-exposure occurring within groups defined by lifetime smoking history (analyzed in the first step) were concentrated in specific groups defined by recent smoking history (analyzed in the second step). This process allowed a detailed understanding of how smoking history moderated the influence of graphic warnings on the intention indices, despite the fact that it was not possible to formally test for the interaction of lifetime smoking history, recent smoking history and experimental condition in a single factorial analysis.

## Results

### Smoking intention

#### First Step: Lifetime Smoking History

A 2 (Experimental Condition: GW Exposure, Control) X 3 (Life History: None, Moderate, Heavy) ANCOVA was performed on post-manipulation smoking intention, treating pre-manipulation smoking intention as a covariate and controlling for age and sex. This revealed statistically significant effects for the pre-manipulation measure of intention, *F* (1, 1158) = 1403.58, *p*<.001, η^2^ = 0.74, and lifetime smoking history, *F* (2, 1158) = 49.89, *p*<.001, η^2^ = 0.20. The nature of the lifetime smoking history effect was that smoking intention increased the more individuals had smoked in the past (no prior experience smoking, *M* = 2.36, *SE* = 0.05; moderate lifetime smoking history, *M* = 2.99, *SE* = 0.05; and heavy lifetime smoking history, *M* = 3.16, *SE* = 0.07). These effects and the remaining covariates accounted for 79 percent of the variance in the criterion, but results also revealed a significant effect of image exposure, *F* (1, 1158) = 4.74, *p* = 0.03, η^2^ = 0.06, such that smoking intention was lower in the GW condition (*M* = 2.77, *SE* = 0.04) than the control (*M* = 2.90, *SE* = 0.05). There was not a significant interaction between lifetime smoking history and the experimental manipulation, *F* (1, 1158) = 2.33, *p* = 0.13, η^2^ = 0.04. However, consistent with predictions of a floor effect, simple main effect analysis revealed there was no effect of GW exposure among those who had never smoked (whose smoking intention stayed relatively low in both conditions), *F*<1. Simple effect analysis also revealed no effect of GW exposure among those who had heavy lifetime smoking history (whose smoking intention stayed relatively high in both conditions), *F*s<1. The effect of GWs on intention occurred only among participants with moderate lifetime smoking history, *F* (1, 1158) = 8.42, *p*<.001, η^2^ = 0.08.

#### Second Step: Recent Smoking History

When simple main effects of experimental condition and recent smoking history was examined within the moderate and heavy lifetime smoking history, simple effects analysis revealed that the effect of the manipulation on smoking intention was statistically significant in one subgroup: those with moderate lifetime smoking history who had also been occasional smokers in the prior 30 days, *F* (1, 312) = 3.67, *p* = 0.05, η^2^ = 0.11. No other subgroups showed changes in smoking intention. This pattern supports the adaptive response hypothesis – GWs did exert beneficial effects on smoking intention, but only among occasional smokers who had not engaged in heavy smoking in the past.

### Quit intention

#### First Step: Lifetime Smoking History

Quit intention was only measured in the (*n* = 631) participants who reported smoking at least once their lifetime (i.e., moderate or heavy lifetime smokers). A 2 (Experimental Condition) X 2 (Life History) ANCOVA was performed on post-manipulation quit intention for this subsample, treating pre-manipulation quit intention as a covariate and controlling for age and sex. This revealed significant effects for the pre-manipulation quit intention, *F* (1, 618) = 147.69, *p*<.001, η^2^ = 0.44, and smoking history, *F* (1, 618) = 4.85 *p* = 0.03, η^2^ = 0.09, such that quit intention was higher among those with heavy lifetime smoking history (*M* = 4.33, *SE* = 0.08) than moderate lifetime smoking history (*M* = 4.09, *SE* = 0.07). In addition, there was a main effect of the experimental manipulation, *F* (1, 618) = 3.89, *p* = 0.05, η^2^ = 0.08, such that quit intentions were higher in the GW condition (*M* = 4.31, *SE* = 0.07) than the control (*M* = 4.11, *SE* = 0.08). This effect did not interact with lifetime smoking history, *p* = 0.25. However, simple main effect analysis revealed that the effect of GW exposure was significant in the group that had smoked 100 or more cigarettes in the past, *F* (1, 618) = 4.81, *p* = 0.03, η^2^ = 0.09, whereas it was not significant among those who had smoked fewer than 100 cigarettes, *F*<1.

#### Second Step: Recent Smoking History

When recent smoking history was examined within the moderate and heavy lifetime smoking groups, simple effects analysis revealed that GW exposure was only statistically significant among those with a heavy lifetime smoking history and who were occasional recent smokers, *F* (1, 303) = 4.57, *p* = 0.03, η^2^ = 0.12. There were no effects among the heavy lifetime smokers who were either currently inactive, *F*<1, or who were daily smokers, *F* (1, 303) = 2.25, *p* = 0.13, η^2^ = 0.09, nor were there effects in any of the groups with moderate lifetime smoking history, *F*s<1. This pattern also supports the adaptive response hypothesis – GWs did exert beneficial effects on quit intention, but only among occasional smokers who had engaged in heavy smoking in the past.

### Individual Differences

Interaction regression methods were applied [62] to determine if any of the individual difference factors (i.e., psychological reactance, self-esteem and sensation seeking) moderated the effect of GW exposure on either of the behavioral intention indices. No statistically significant interaction effects were observed.

## Discussion

### Principal Findings

GW exposure was found to lower the intention to smoke in young adults with a moderate lifetime smoking history (between 1 and 100 cigarettes) and raise the quit intentions in those with a heavy lifetime smoking history (100 or more cigarettes), with each effect concentrated in the subsample of occasional smokers (who had smoked some but not all of the previous 30 days). No evidence was found to suggest defensive “boomerang” effects on intention, either for those with more extensive smoking histories or for those with high scores on individual difference measures linked to defensive processing of risk-prevention messages (e.g., psychological reactance). These findings support the adaptive-responding hypothesis and challenge the defensive-responding hypothesis, suggesting that even the young, occasional smokers who might be predicted to react defensively to the graphic nature of antismoking tobacco warnings instead responded with diminished interest in smoking in the future.

### Strengths and Weaknesses

The primary contributions of the current study are its attention to behavioral intention as an outcome and its consideration of individual difference factors that might alter the influence of GWs on this outcome. The effects of GWs on intention that we observed reinforce findings from (1) naturalistic studies documenting decreases in smoking rates in countries that mandate GWs on cigarette packaging and (2) other laboratory studies showing beneficial effects of GW exposure on health-related cognitions. In so doing, they may bolster claims of a government interest in mandating GWs on cigarette packaging in the US [2]. In addition, our attention to individual difference factors addresses a limitation of many past studies, in that it identified two groups of young smokers who might be especially responsive to GWs: (1) occasional smokers who are still in an early, “experimental” stage with cigarettes, and (2) occasional smokers who have adopted a more stable pattern of occasional use.

As with any study, however, there are limitations to the current investigation. Most critically, exposure to GWs was carried out online, and only state changes in intentions were assessed. As Hammond, Driezen and Bourdreau (2013) note, “there is no way to replicate ‘real world’ exposure to health warnings in an experimental study” and we acknowledge this limitation with the current investigation [63] (p. 100). The current results also are limited because, although they advance our understanding by pointing to potential moderators of GWs on behavior, more exhaustive studies that track actual behavior change over time will be needed before stronger conclusions can be made. Our study was further limited in that it did not utilize probability-based sampling methods. Investigations utilizing larger and more representative samples might uncover defensive reactions in subgroups that our study failed to represent in sufficient numbers. Finally, the current study investigated the influence of the GWs that were part of the original FDA effort. If the FDA again seeks to mandate GWs, it will need to investigate reactions to the new labels it develops. The current study introduces a methodology that might be of use in these efforts; one that models change in behavioral intention and the moderating effects of individual difference factors.

### Implications

Despite these limitations, the findings are suggestive of ways to maximize the influence that GWs exert on young adults. Concentration of effects among the occasional smokers points to the need for greater understanding of these individuals. Edwards and colleagues (2010) followed a sample of occasional smokers, aged 18 and older. Their group was comparable to the subsample in our study that had a heavy lifetime smoking history and an occasional recent smoking history (i.e., all had smoked at least one cigarette in the past 30 days and 100 cigarettes in their lifetime) [37]. They found that members of this group were at considerable risk of continuing to smoke (as 45% remained occasional smokers at one-year follow up) or of increasing their smoking (as 17% transitioned to becoming daily smokers). Perhaps because occasional smokers perceive their behavior as low-risk, intention to quit was lower in this group than in a comparison group of daily smokers. It is thus encouraging that in the current study, GWs influenced the quit intentions of the occasional smokers who had smoked more than 100 cigarettes in their lifetime. As Edwards and colleagues point out, however, there is great heterogeneity among occasional smokers – not just in terms of past smoking history – but also perceived physical addiction and intentions to quit at some point in the future [32]. Our findings suggest that occasional smokers are responsive to GW messages, whether they have moderate or heavy lifetime smoking histories. These two groups only differed in whether they reacted to the GWs in this study with a decreased willingness to smoke in the future if opportunities arise or an increased intention to quit. One interpretation of this difference is that occasional smokers who are still in an early stage with cigarettes might not perceive their diminished interest in smoking as an intention to “quit,” per say, as they do not yet think of themselves as smokers. In contrast, occasional smokers who have adopted a more stable pattern of occasional use might appreciate that they have become smokers, and so they frame this cognitive change in terms of a quit intention.

Regardless of the meaning of these shifts, more should be known about the factors that shape the ways that occasional smokers react to and processes the dangers of smoking that can be communicated by GWs. This understanding might lead to better message targeting of messages to this most-responsive of groups. For instance, Brown, Carpenter and Sutfin (2011) conducted focus groups on a sample of 18–25 year olds who had smoked occasionally for a stable period of at least 6 months [64]. They found that, although many recognize the general risks of smoking, the group as a whole tended to minimize the health and addiction risks of their actions. Given the current finding that this same group will be most strongly influenced by GWs, there may be benefit to targeting their misperceptions with GWs. This would mean designing GWs that focus more on the dangers of “light” or occasional smoking than was done in the original FDA effort. Six of the 9 GWs in that campaign drew attention to the long-term effects of smoking; risks that might not be perceived as relevant among occasional smokers (e.g., risks of cancer, heart diseases and death). Two other messages focused on the dangers of smoke to babies and children and one noted the benefit of quitting. Had the GWs targeted the belief structure underlying occasional smoking, the effects might have been stronger than was observed in the current study. Ultimately, this is an empirical question and one deserving of future investigations. Research should determine if occasional smokers are more influenced by GWs that focus attention on extreme health risks (that many may believe to be more relevant for heavy smokers) or on dispelling some of their misperceptions about the risks of what they perceive to be relatively low-risk smoking.
